# Microtubule Stabilization Enhances the Chondrogenesis of Synovial Mesenchymal Stem Cells

**DOI:** 10.3389/fcell.2021.748804

**Published:** 2021-10-20

**Authors:** Jiawei Li, Ziying Sun, Zhongyang Lv, Huiming Jiang, Anlong Liu, Maochun Wang, Guihua Tan, Hu Guo, Heng Sun, Rui Wu, Xingquan Xu, Wenjin Yan, Qing Jiang, Shiro Ikegawa, Dongquan Shi

**Affiliations:** ^1^State Key Laboratory of Pharmaceutical Biotechnology, Division of Sports Medicine and Adult Reconstructive Surgery, Department of Orthopedic Surgery, Nanjing Drum Tower Hospital, The Affiliated Hospital of Nanjing University Medical School, Nanjing, China; ^2^Department of Sports Medicine and Adult Reconstructive Surgery, The Affiliated Nanjing Hospital of Nanjing Medical University, Nanjing, China; ^3^Laboratory for Bone and Joint Diseases, RIKEN Center for Integrative Medical Science (IMS, RIKEN), Tokyo, Japan

**Keywords:** microtubule stabilization, synovial mesenchymal stem cells, chondrogenesis, Yes-associated protein, primary cilia

## Abstract

Mesenchymal stem cells (MSCs) are well known for their multi-directional differentiation potential and are widely applied in cartilage and bone disease. Synovial mesenchymal stem cells (SMSCs) exhibit a high proliferation rate, low immunogenicity, and greater chondrogenic differentiation potential. Microtubule (MT) plays a key role in various cellular processes. Perturbation of MT stability and their associated proteins is an underlying cause for diseases. Little is known about the role of MT stabilization in the differentiation and homeostasis of SMSCs. In this study, we demonstrated that MT stabilization via docetaxel treatment had a significant effect on enhancing the chondrogenic differentiation of SMSCs. MT stabilization inhibited the expression of Yes-associated proteins (YAP) and the formation of primary cilia in SMSCs to drive chondrogenesis. This finding suggested that MT stabilization might be a promising therapeutic target of cartilage regeneration.

## Introduction

Articular cartilage locates at the end surface of the bone and is responsible for the load-bearing as well as lubricating the friction in the joint. Cartilage exhibits poor ability to spontaneously repair due to its aneural and avascular characteristics (Huey et al., 2012). Current strategies for cartilage repair, including microfracture, autologous chondrocyte implantation (ACI), and matrix-induced autologous chondrocyte implantation, are unable to reemerge the biomechanical properties of cartilage (Vasara et al., 2005; Gille et al., 2010). Although a variety of attempts to manipulate stem cells and tissue engineering scaffolds have been made, there is still no ideal therapy to repair articular cartilage (Shi et al., 2016; Chuah et al., 2017; Li et al., 2019). Mesenchymal stem cells (MSCs) are well known for their multi-directional differentiation potential to chondrocytes, adipocytes, and osteocytes (Gale et al., 2019). Previous researches mainly focused on bone marrow–derived MSC (BM-MSC). However, bone marrow is a kind of tissue that is difficult to obtain and the quantity is also insufficient, which limits the wide application of BM-MSCs in the clinic. The cells isolated from the synovium of human knee joints exhibited the stem cell characteristics, which were named synovial mesenchymal stem cells (SMSCs) (De Bari et al., 2001). Besides, SMSCs have been attracted a lot of attention because they had more accessible sources, a high proliferation rate, low immunogenicity, and greater chondrogenic differentiation potential (Li et al., 2020). Based on these, current studies demonstrated the promising therapeutic effect of SMSCs in cartilage and bone disease. However, the differentiation of SMSCs is affected by a variety of biological factors and signaling pathways. Therefore, exploring how to regulate the differentiation of SMSCs is a vital factor to effectively play its role.

The cytoskeleton is a three-dimensional, highly organized network comprising actin, intermediate filament, and microtubule (MT) (Moujaber and Stochaj, 2020). They are kind of a driver and a regulator of signaling pathways that sense and transduce a variety of cellular information involved in mechano-transduction. An intact intermediate filament network played a key role in the maintenance of the chondrocyte phenotype and chondrogenesis (Blain et al., 2006). In addition, the actin organization of SMSCs was involved in the regulation of transforming growth factor-β1 (TGF-β1) and SMAD pathway to induce chondrogenesis (Xu et al., 2012). As for MT, it involves various cellular processes, including maintenance of cell shape, motility, transport, and interactions with extracellular matrix (ECM). MT is assembled with heterodimers of α- and β-tubulin to form a long hollow polymer. MT is constantly remodeled through alternating growth and shrinkage of their extremities (Borisy et al., 2016). The status of MT (dynamic instabilized or stabilized) helps to decide cell shape and organization and acts as a substrate for motor-driven intracellular transport. Stable MT undergoes the consistent polymerization or growth, but without drastic catastrophe (Goodson and Jonasson, 2018). Perturbation of MT stability and their associated proteins was an underlying cause for diseases such as Alzheimer’s disease and various cancers (Ilan, 2018). However, little is known about the role of MT stabilization in the differentiation and homeostasis of SMSCs.

Yes-associated protein (YAP), an important mechano-transduction protein, is an effector in the Hippo signaling pathway and is regulated by cytoskeleton remodeling (Deng et al., 2018; Elbediwy and Thompson, 2018). It was reported that YAP suppressed chondrogenic differentiation (Karystinou et al., 2015; Deng et al., 2016). Moreover, primary cilium is the key mechano-signaling sensor in chondrocytes, which mainly consists of MTs (Ruhlen and Marberry, 2014), and is where the complex IHH signaling pathway is anchored (Bangs and Anderson, 2017). Previous studies demonstrated that primary cilium was critical for regulating cytoskeleton organization and YAP activity (Ruhlen and Marberry, 2014; Kim et al., 2015). However, the mechanism that regulates YAP activity and primary cilium in the context of MT stabilization to influence the chondrogenesis of SMSCs remains to be elucidated.

In this study, we examined the role of MT stabilization in chondrogenic differentiation of SMSCs by using docetaxel (MT stabilizer) and nocodazole (MT destabilizer). We demonstrated that MT stabilization by docetaxel treatment maintained cartilage functions and promoted chondrogenesis of SMSCs. MT stabilization inhibited YAP activity and ciliogenesis in SMSCs to drive chondrogenic differentiation. Our results indicate that MT stabilization would be a potential therapeutic target for promoting articular cartilage repair.

## Materials and Methods

### Ethics Statement

The collection of human synovium and experimental protocols were approved by the Ethical Committee of the Nanjing Drum Tower Hospital, the Affiliated Hospital of Nanjing University Medical School (2009022). All experimental procedures followed the guidelines established by the Declaration of Helsinki (General Assembly of the World Medical Association, 2014).

### Human Samples

Human OA synovium samples were collected from seven OA patients (56–78 years old; Kellgren–Lawrence grade IV) when they underwent total knee arthroplasties. All of them were processed to establish the primary cultures of SMSC.

### Cell Culture

Human OA synovium samples (*n* = 7) were processed to establish primary cell cultures. To establish SMSC culture, the synovium fragments were subsequently lysed with 0.2% collagenase I in DMEM/F12 at 37°C for 6 h. After filtration and centrifugation, the synovium internal cellular mass was placed in a chondrogenic medium (CM). The cells were cultivated in a humidified environment at 37°C and 5% CO_2_ followed by regular replacement of the culture medium every 2 days.

### Flow Cytometric Analysis

The cell samples of SMSCs were collected at the second passage and then washed with 5% bovine serum albumin three times. The cells were distributed approximately at 500,000 cells per tube. After centrifugation (500 × *g*, 5 min), the pellets were resuspended and incubated with the 100 μl solution containing different antibodies [CD68-FITC (#562117-BioLegend, United States)/CD90-APC (#561971-BioLegend), and CD34-PE (#560941-BioLegend)/CD105-APC (#562408-BioLegend)] or 100 μl blocking solution as negative control for 30 min under the temperature at 37°C. Then, the samples were washed with phosphate buffered saline (PBS) three times and analyzed to BD AccuriC6 Plus Flow Cytometer (BD, Biosciences, United States). Data were further analyzed by the FlowJo software.

### 5-Ethynyl-2′-Deoxyuridine Assay

The EdU Reagent Kit (Ribobio, Guangzhou, China) was used to investigate cell proliferation according to the manufacturer’s instructions. After being treated with nocodazole and docetaxel for 1 week, the SMSCs were incubated with 10 μM EdU for 2 h. The images were obtained with a fluorescence microscope (Zeiss, Heidelberg, Germany).

### Cell Counting Kit-8 Assay

The cell viability of SMSCs was assessed by CCK8 assay (Dojindo, Japan, Kumamoto) according to the manufacturer’s instructions. After being washed with PBS, the cells in each well were incubated with DMEM/F12 comprising 10% (v/v) CCK-8 solution at 37°C for 2 h. The absorbance was measured at 450 nm with a microplate reader (Thermo Scientific, Logan, UT, United States).

### Multiple Differentiation Induction of Synovial Mesenchymal Stem Cells

#### Chondrogenesis

The SMSCs were cultivated in chondrogenic medium, which consisted of 10 ng/ml TGF-β1 (Peprotech, New Jersey, United States), 50 μg/ml L-ascorbic acid 2-phosphate (Aladdin, Shanghai, China), 100 mM sodium pyruvate (Aladdin), 40 μg/ml proline (Aladdin), 10^–7^ M dexamethasone (Sigma, Missouri, United States), 2% fetal bovine serum (FBS, Gibco, MA, United States), 1% penicillin–streptomycin (Gibco), and ITS + premix (Sigma) in DMEM-HG (4.5 g/L; Gibco) basic medium. After 14 days of culture, the cells were fixed in 4% paraformaldehyde for 30 min, and then safranin O staining (SO) was performed.

#### Osteogenesis

The SMSCs was cultivated in osteogenic medium, which consisted of 50 μg/ml L-ascorbic acid 2-phosphate (Sigma), 10^–7^ M dexamethosone (Sigma), 10 mM β-glycerophosphate (Sigma), 2 mM glutamine (Gibco), 110 μg/ml sodium pyruvate (Gibco), 10% fetal bovine serum (FBS, Gibco), and 1% penicillin–streptomycin (Gibco) in DMEM-HG (4.5 g/L; Gibco) basic medium. After 14 days of culture, the cells were fixed in 4% paraformaldehyde for 30 min and then alizarin red S (ARS) staining was performed.

#### Adipogenesis

The SMSCs was cultivated in adipogenic growth medium for 2 days, which consisted of 10 μg/ml insulin, 10^–7^ M dexamethasone, 0.5 mM isobutylmethylxanthine, 10% FBS (Gibco), and 1% penicillin–streptomycin (Gibco) in DMEM-HG (4.5 g/L; Gibco) basic medium. After 2 days, the cells were kept in medium supplemented with 10 μg/ml insulin for 2 days. Then the culture medium was replaced with fresh DMEM-HG containing 10% FBS for 2–3 days. Then the cells were fixed in 4% paraformaldehyde for 30 min and then Oil red O staining was performed.

### Microarray Data Collection and Processing

A gene expression profile (GSE82107) was downloaded from the Gene Expression Omnibus (GEO) database.^1^ The microarray dataset was based on HGU133plus2.0 platform and contained synovium from 10 OA donors and 7 healthy donors. Raw data were normalized by robust multi-array average algorithm. There were 54,675 probes and combined into 20,161 gene symbols. Inflammatory genes and stem cell–related genes were extracted and filtered by | log(fold change)| > 1.25 with *p*-value < 0.05. Heatmap and GO dotplot were conducted with R pheatmap (v1.0.12) and gglpot2 (v3.32) packages.

### Quantitative Real-Time PCR

Cellular mRNA was isolated from SMSCs using TRIzol reagent (Thermo Fisher Scientific, Logan, UT, United States). Complementary DNA (cDNA) was synthesized from mRNA using reverse transcription reagents (Vazyme Biotech, Nanjing, China) and quantitative PCR assays were carried out using a LightCycler 480 II (Roche Molecular Biochemicals, Indianapolis, IN, United States). The primer sequences are listed in Supplementary Material.

### Western Blot Analysis

Proteins were extracted from the cells using RIPA lysis buffer supplemented with 1 mM phenylmethanesulfonyl fluoride and 1 mM protein phosphatase inhibitor. After lysis, samples were centrifuged for 10 min at 12,000 rpm at 4°C. The protein concentration of the samples was determined by the BCA protein assay kit (Thermo Scientific). Proteins from the prepared lysates were then separated on 10% (w/v) SDS-polyacrylamide gels and transferred onto a polyvinylidene fluoride membrane (Bio-Rad, Hercules, CA, United States). The membranes were blocked with 5% (w/v) milk (Bio-Rad) for 2 h at room temperature and then incubated overnight at 4°C with primary antibodies for either Ace-tubulin (1:1,000; #5335s-Cell Signaling Technology, Boston, United States), Col II (1:5,000; #ab34712-Abcam, Cambridge, United Kingdom), SOX9 (1:1,000; #82630s-Cell Signaling Technology), SMAD3 (1:1,000; #9513s-Cell Signaling Technology), phosphorylated SMAD3 (1:1,000; #9520s-Cell Signaling Technology), phosphorylated YAP (1:1,000; #4911s-Cell Signaling Technology), YAP (1:1,000; #4912s-Cell Signaling Technology), TAZ (1:1,000; #4883s-Cell Signaling Technology), IFT88 (1:1,000; #60227-1-lg-Proteintech, Wuhan, China), SMO (1:1,000; #66851-1-lg-Proteintech), GLI1 (1:1,000; #66905-1-lg-Proteintech), and GAPDH (1:1,000; #5174s-Cell Signaling Technology). The membranes were then washed using TBS with 0.05% Tween 20 (TBST) three times and incubated with horseradish peroxidase-conjugated secondary antibodies for 60 min. All protein signals were detected using a ChemiDocXRS + Imaging System (Tanon, Shanghai, China). All experiments were repeated five times.

### Cell Immunofluorescence

Cells were washed with PBS, fixed in 4% paraformaldehyde, and permeated in 0.1% Triton X-100 for 15 min. After blocking with 5% bovine serum albumin for 1 h at 37°C, the cells were incubated with primary antibodies overnight at 4°C. The cells were then washed with PBS and incubated with FITC or TRITC conjugated second antibodies for 1 h at 37°C and labeled with DAPI for 7 min. Twenty fields from each slide were chosen randomly for observation with a fluorescence microscope (Zeiss).

### Pellet Cultures and Micro-Mass Cultures

A pellet consisting of 5 × 10^5^ cells was cultured in a microfuge tube for 4 weeks in a humidified environment at 37°C and 5% CO_2_ with chondrogenic differentiation medium. The medium was supplemented with 10 ng/ml TGF-β1 (Peprotech) and 500 ng/ml BMP2 (Peprotech). The medium was refreshed twice per week.

For micro-mass culture, 5 × 10^5^ cells were seeded at the center of the 24-well plate avoiding touching the sides of the wells. The cells were cultivated in a humidified environment at 37°C and 5% CO_2_ with chondrogenic differentiation medium. The medium was supplemented with 10 ng/ml TGF-β1. The medium was refreshed twice per week.

### Histological and Microscopy Analysis

The micro-mass of SMSCs were fixed in 4% (v/v) paraformaldehyde for 15 min, and after being washed by phosphate buffer saline (PBS), crystal violet staining, SO staining, and alcian blue staining (AB) were performed on the specimens according to the manufacturer’s instructions. The pellets were fixed in 4% (v/v) paraformaldehyde for 1 day, and after dehydration, the specimens were embedded in paraffin and cut into 3-μm coronal sections. Sections of each tissue were then processed and stained with SO and toluidine blue staining (TB). Photomicrographs of results were captured with a microscope (Zeiss, Heidelberg, Germany).

### Immunohistochemical Staining and Immunofluorescent Analysis

Immunohistochemical staining and immunofluorescent analysis were performed according to the manufacturer’s instructions. Serial sections were incubated with primary antibodies for Col II (1:500; #ab34712-Abcam) and Col I (1:300; #BA0325-Boster, Wuhan, China) overnight at 4°C. For immunohistochemical staining, HRP conjugated secondary antibodies were added to the slides and incubated at 37°C for 1 h. For immunofluorescent staining, FITC or TRITC conjugated secondary antibodies were added to the slides and incubated at room temperature for 1 h in the dark. Photomicrographs of sections were captured with a fluorescence microscope (Zeiss).

### Short Interfering RNA Transfection

siRNA against the human *YAP1* gene was designed and synthesized as the following sequence: 5-GGUGAUACUAUCAACCAAATT-3 (Hippobio). SMSCs were seeded in 6-well plates and grown to approximately 70% confluence. Cells were then transfected with either 50 nM siRNA-YAP or negative control in Lipofectamine 3000 (Thermo Fisher Scientific) for 12 h according to the manufacturer’s instructions.

### Statistical Analysis

All data were expressed as means ± SEMs. Statistical analysis was performed with one-way ANOVA using GraphPad Prism 8 for Windows. Differences were considered statistically significant when *p* < 0.05.

## Results

### Characterization and Differential Potency of Synovial Mesenchymal Stem Cells

**FIGURE 1:**
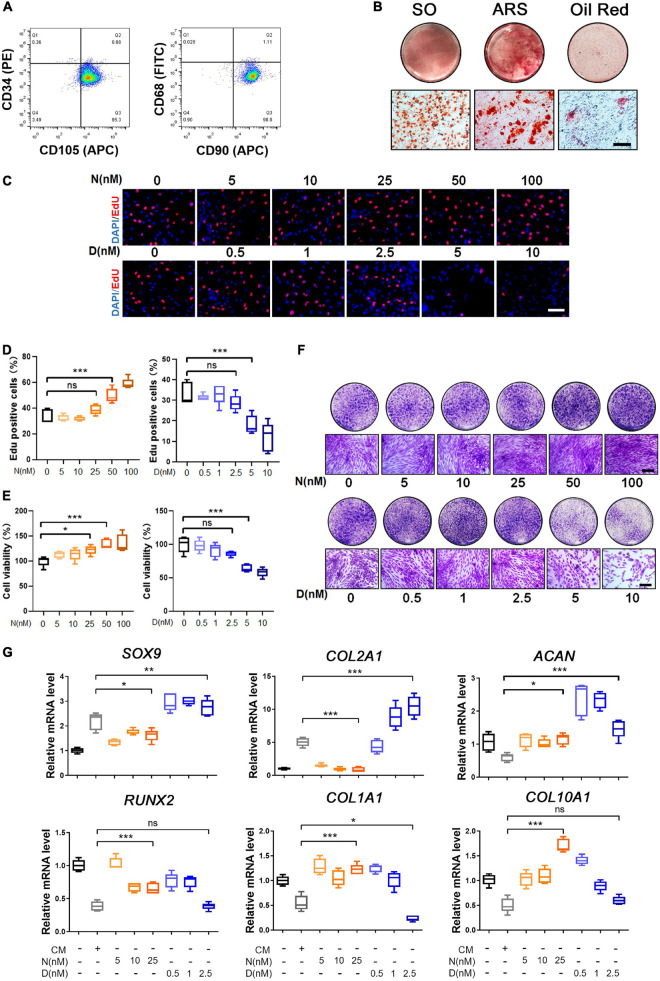
Characterization and trilineage differential potency of SMSCs from osteoarthritis (OA) keen synovium. **(A)** Surface marker expression of synovial mesenchymal stem cells (SMSCs). Results showed negativity for hematopoietic marker (CD34) and macrophage marker (CD68) and showed positivity for mesenchymal stem cell (MSC) markers like CD105 and CD90. **(B)** Multi-directional differentiation of SMSCs; chondrogenesis was accessed by safranin O staining (SO), osteogenesis was accessed by alizarin red S staining (ARS), and adipogenesis was accessed by Oil red O staining. **(C)** The EdU staining of SMSCs after the treatment of different concentrations of nocodazole (N) (5, 10, 25, 50, 100 nM) and docetaxel (D) (0.5, 1, 2.5, 5, 10 nM) in chondrogenic medium (CM) for 1 week. Scale bar, 100 μm. **(D)** Quantification of the data of **(C)**. *n* = 5. **(E)** The cell viability of SMSCs after the treatment of different concentrations of nocodazole (N) (5, 10, 25, 50, 100 nM) and docetaxel (D) (0.5, 1, 2.5, 5, 10 nM) in chondrogenic medium (CM) for 1 week. **(F)** Crystal violet staining of SMSCs after the treatment of different concentrations of nocodazole (N) (5, 10, 25, 50, 100 nM) and docetaxel (D) (0.5, 1, 2.5, 5, 10 nM) in chondrogenic medium (CM) for 1 week. Scale bar, 100 μm. **(G)** RT-qPCR analyses of *SOX9*, *COL2A1*, *ACAN*, *RUNX2*, *COL1A1*, and *COL10A1* in SMSCs treated with nocodazole (N) and docetaxel (D) in chondrogenic medium (CM) for 1 week. 2.5 nM of docetaxel had significant effects on chondrogenesis in SMSCs. Data are represented as the mean ± SEM. *p < 0.05, **p < 0.01, ***p < 0.001.

First, to harvest SMSCs, we isolated the cells from the human knee joint synovium after total knee arthroplasty and cultured them to the second passage (P2). The characterization of surface epitopes on SMSCs (P2) was performed and revealed satisfactory stem cell potency (Figure 1A). The results of flow cytometry showed that the cells were negative for the hematopoietic marker (CD34) and macrophage marker (CD68) while positive for MSC markers such as CD105 and CD90. To verify the multi-differential potency of the isolated synovial cells, we cultured the cells with chondrogenic, osteogenic, and adipogenic medium, respectively, to induce trilineage differentiation. The results of histological analysis, including Safranin O staining (SO), ARS staining, and Oil red O staining, elucidated the expected multi-differential capacity of SMSCs (Figure 1B). Moreover, mRNA expressions of inflammatory genes and stem cell–related markers in knee joint synovium were compared between OA and healthy donors in a public data. The expression of matrix metalloproteinases (MMPs), including *MMP1*, *MMP2*, *MMP3*, *MMP9*, and *MMP13*, were increased in OA synovium. However, the genes in stem cell–related markers, including *LY6E*, *NT5E* (CD73), and *THY1* (CD90), were also increased in OA synovium (Supplementary Figure 1A). The biological processes of ECM organization, extracellular structure organization, and collagen metabolism were enriched (Supplementary Figure 1B).

### Microtubule Stabilization Promotes Chondrogenesis of Synovial Mesenchymal Stem Cell

Nocodazole and docetaxel were used to destabilize and stabilize microtubule (MT), respectively. To determine the optimal drug concentration, we performed subsequent experiments to assess the proliferation and cell viability of SMSCs that were treated with different concentrations of nocodazole and docetaxel. First, the results of EdU staining revealed that the proliferation of SMSCs was not significantly affected with the treatment of nocodazole (5, 10, and 25 nM) and docetaxel (0.5, 1, and 2.5 nM), while it was significantly enhanced with a higher concentration of nocodazole (50 and 100 nM) and inhibited with a higher concentration of docetaxel (5 and 10 nM) (Figures 1C,D). Cell viability of SMSCs was assessed by CCK-8 assay. The results indicated that 25 nM nocodazole had a slight enhancement in the proliferation of SMSCs, while docetaxel had no significant effect on the cell viability within 2.5 nM (Figure 1E). The crystal violet staining demonstrated a similar result with EdU staining and CCK8 assay (Figure 1F). To avoid the impact of excessive differences in cell proliferation, nocodazole within 25 nM and docetaxel within 2.5 nM were chosen for further investigation of the effect of MT stabilization on the differentiation of SMSCs. Compared with the chondrogenic medium (CM) group, RT-PCR analysis revealed that MT stabilization (docetaxel treated) upregulated the mRNA expression of *SOX9* and *COL2A1* and decreased the level of *COL1A1* (Figure 1G). However, the nocodazole treatment inhibited the mRNA expression of *SOX9* and *COL2A1* (Figure 1G). Besides, the expression of *RUNX2* and *COL10A1* was unchanged when the concentration of docetaxel was 2.5 nM (Figure 1G).

**FIGURE 2:**
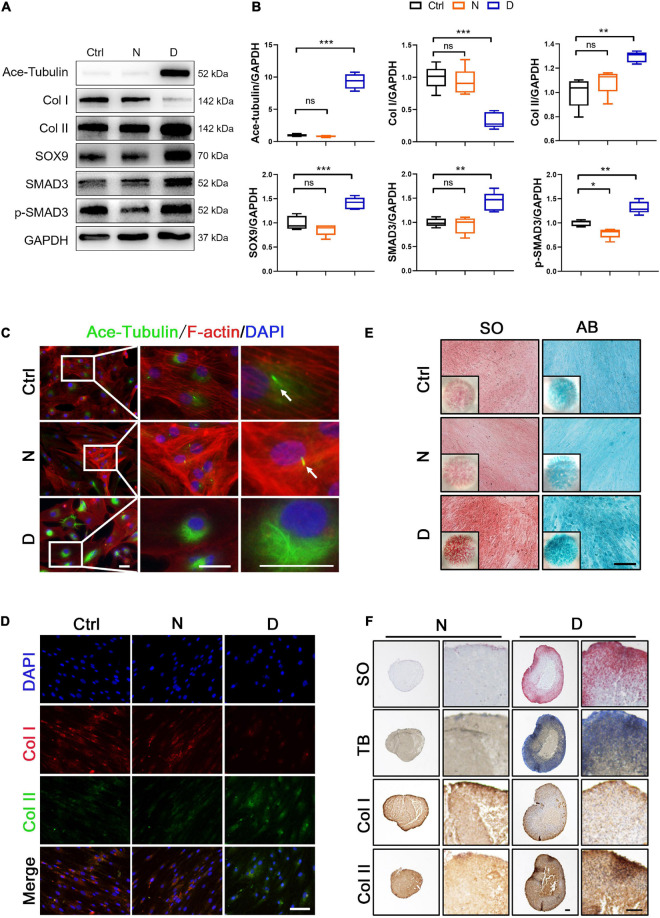
The effect of microtubule (MT) stabilization in the chondrogenesis of SMSCs. **(A)** Western blot analysis of Ace-Tubulin, Col I, Col II, SOX9, SMAD3, and phosphorylated SMAD3 expression in synovial mesenchymal stem cells (SMSCs) treated with nocodazole (N) and docetaxel (D) in chondrogenic medium (CM) for 1 week. **(B)** Quantification of the data of **(A)**. *n* = 5. **(C)** Immunofluorescence staining of Ace-Tubulin and F-actin. Scale bar, 50 μm. The docetaxel treatment increased the expression level of Ace-Tubulin. **(D)** Co-immunofluorescence staining of Col I and Col II. Scale bar, 100 μm. **(E)** Safranin O staining (SO) and Alcian blue (AB) staining of SMSC micro-mass culture grown in chondrogenic medium (CM) treated with nocodazole (N) and docetaxel (D) for 4 weeks. **(F)** pellet cultures of SMSCs in chondrogenic medium (CM) treated with nocodazole and docetaxel for 4 weeks. SO staining, toluidine blue (TB) staining, and immunohistochemical staining of Col II and Col I were performed to access the chondrogenesis of SMSC pellets. Scale bar, 100 μm. Data are represented as the mean ± SEM. *p < 0.05, **p < 0.01, ***p < 0.001.

Based on the data of the RT-PCR analysis, 25 nM nocodazole and 2.5 nM docetaxel were chosen for the treatment of SMSCs under CM for 2 weeks. Acetylated tubulin (Ace-Tubulin) was the marker of stabilized MT because it was demonstrated that this kind of tubulins was characterized as long-lived, polymerized, and stable MTs (Westermann and Weber, 2003). In the results of western blot and immunofluorescence staining, the expression of Ace-Tubulin was enhanced in SMSCs treated with docetaxel (Figures 2A–C). As the MT stability increased, the expression of Col II, SOX9, and the activity of SMAD3 was upregulated, and Col I was decreased at the protein level (Figures 2A,B). The ratio of Col II to Col I also was improved by the treatment of docetaxel, which was confirmed by co-immunofluorescence staining (Figure 2D). The micro-mass and pellet cultures were performed to further verify the chondrogenesis of SMSCs in 2D and 3D environment by MT stabilization. The results of SO staining and alcian blue (AB) staining in the SMSC micro-mass culture exhibited a stronger chondrogenic effect of docetaxel treatment when compared with nocodazole and control groups (Figure 2E). Moreover, the positive results in SO staining, toluidine blue staining (TB), and immunohistochemical staining for Col II and negative for Col I revealed that more cartilage-like ECM was formed in the pellet that was treated with docetaxel compared with the CM control and nocodazole treatment groups (Figure 2F).

### Microtubule Stabilization Inhibits Yes-Associated Proteins Expression and Activity in Synovial Mesenchymal Stem Cell

**FIGURE 3:**
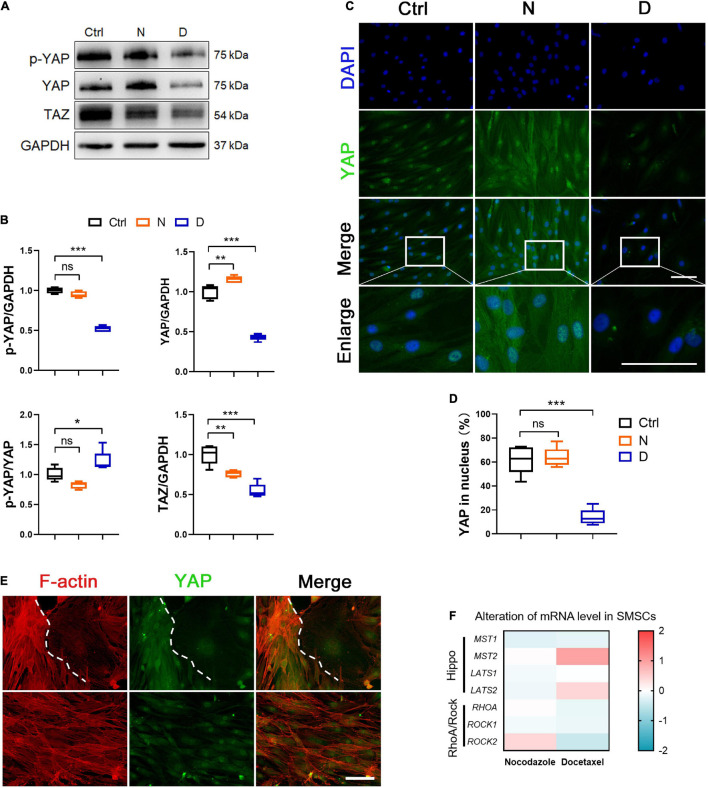
The effect of microtubule (MT) stabilization in YAP expression in SMSCs. **(A)** Western blot analysis of phosphorylated Yes-associated protein (YAP), total YAP and TAZ expression in synovial mesenchymal stem cells (SMSCs) treated with nocodazole (N) and docetaxel **(D)** in chondrogenic medium (CM) for 1 week. **(B)** Quantification of the data of **(A)**. *n* = 5. **(C)** Immunofluorescence staining for YAP of SMSCs. Scale bar, 100 μm. **(D)** Quantification of YAP in nucleus. *n* = 5. MT stabilization inhibited the YAP expression in SMSCs. **(E)** Co-immunofluorescence staining images of F-actin and YAP in SMSCs without docetaxel or nocodazole treatment (upper panel: view 1; lower panel: view 2). Scale bar, 200 μm. **(F)** RT-qPCR analysis of *MST1*, *MST2*, *LATS1*, *LATS2*, *RHOA*, *ROCK1*, and *ROCK2* in SMSCs treated with either nocodazole (N) or docetaxel (D) for 1 week. Data are represented as the mean ± SEM. *p < 0.05, **p < 0.01, ***p < 0.001.

YAP plays an important role in sensing and transducing the signal from mechanical stimuli and cytoskeleton remodeling. When YAP was inactivated, it was transported to the outside of the nucleus and phosphorylated. We investigated the phosphorylation and expression level of YAP, and its paralog transcriptional co-activator (TAZ) in SMSCs to demonstrate whether MT stabilization was involved in the regulation in YAP. Western blot analyses showed that both total and phosphorylated YAP in SMSCs were significantly decreased, while the ratio of phosphorylated YAP in total increased by docetaxel treatment compared with the control group (Figures 3A,B). Besides, MT destabilization by nocodazole treatment promoted the total expression of YAP (Figures 3A,B). The expression of TAZ both decreased by docetaxel and nocodazole treatment in SMSCs (Figures 3A,B). In the image of immunofluorescence, the nocodazole treatment significantly increased total YAP, while it was hardly observed in both nucleus and cytoplasm after the docetaxel treatment (Figures 3C,D).

It has been reported that triggering F-actin polymerization and stress fiber formation promotes YAP/TAZ activity (Dupont et al., 2011), while treatment with cytochalasin D, a reagent that promoted actin filament depolymerization, induced cytoplasmic re-localization of YAP (Kim et al., 2015). Our findings in SMSCs revealed that the nocodazole treatment not only increased the polymerization of F-actin but also increased the expression and nuclear re-localization of YAP (Figures 2C, 3A), which was consistent with other findings regarding the role of YAP/TAZ in mechano-transduction for sensing cytoskeletal tension (Dupont et al., 2011). Moreover, we found that F-actin was depolymerized when SMSCs were treated with docetaxel, which was similar with the effect reported for cytochalasin D (Figure 2C), and YAP expression was diminished in both cytoplasm and nucleus (Figure 3A). We captured two interesting views of YAP and F-actin co-immunofluorescence in the control SMSC group (Figure 3E). YAP was enriched in the area where F-actin was highly polymerized, and weak cytoplasmic YAP was observed where F-actin was depolymerized (upper panels). The YAP expression was stable when the status of F-actin was fluctuating between polymerization and depolymerization (lower panels). Furthermore, we investigated the components of upstream signaling components from YAP, including the negative regulative Hippo pathway (*MST1/2*, *LATS1/2*) and the positive regulative Rho pathway (*RHOA*, *ROCK1/2*). The mRNA levels of *MST2* and *LATS2* were upregulated and those of *RHOA*, *ROCK1*, and *ROCK2* were diminished in SMSCs with the docetaxel treatment compared with the control group (Figure 3F). These results illustrated that MT stabilization in SMSCs inhibited the YAP expression via actin depolymerization and repressed the YAP activity via regulating Hippo pathway and Rho pathway.

### Microtubule Stabilization Restrains the Formation of Primary Cilia in Synovial Mesenchymal Stem Cell

**FIGURE 4:**
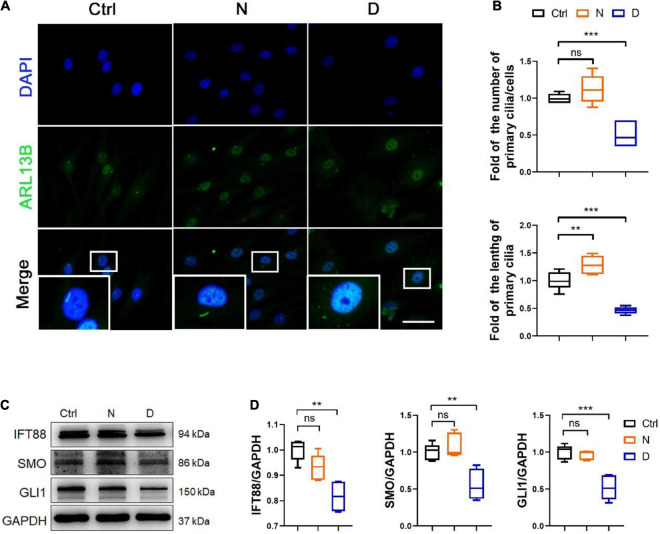
The inhibition of microtubule (MT) stabilization in the formation of primary cilia in SMSCs. **(A)** Immunofluorescence staining of ARL13B (for representing primary cilia) in synovial mesenchymal stem cells (SMSCs) treated with nocodazole (N) and docetaxel (D) in chondrogenic medium (CM) for 1 week. **(B)** Quantification of **(A)** for the number and length of primary cilia in SMSCs. *n* = 6. **(C)** Western blot analysis of IFT88, SMO, and GLI1 in SMSC. **(D)** Quantification of the data of **(C)**. *n* = 5. Data are represented as the mean ± SEM. **p < 0.01, ***p < 0.001.

The Ace-Tubulin-rich small rod-like structures were observed in co-immunofluorescence staining for F-actin and Ace-tubulin in SMSCs, which represented primary cilia (Figure 2C). The primary cilium is a special structure that plays a key role in sensing biomechanical signal, transporting substance between cell membrane, and regulating several signaling pathways, including the hedgehog (HH) pathway (Ruhlen and Marberry, 2014; Bangs and Anderson, 2017). Because MT consists of the main component of primary cilium, we investigated the effect of MT stabilization in primary cilia formation and the Indian hedgehog pathway (IHH) in the chondrogenic differential process of SMSCs. We performed the immunofluorescence staining for ARL13B (a marker for primary cilia within the ADP-ribosylation factor) (Larkins et al., 2011) to investigate the length and quantity of primary cilia. As shown in the image of Figures 4A,B, the length and number of primary cilia were both inhibited with the docetaxel treatment in SMSCs, when compared with the nocodazole treatment group. The western blot results revealed that the docetaxel treatment markedly reduced the expression levels of IFT88 (a protein involved in the transport process in cilia) (Wang et al., 2016), SMO, and GLI1 (member proteins of IHH) (Briscoe and Therond, 2013), similar to its effect on ciliogenesis (Figures 4C,D). These findings indicated that MT stabilization had a negative effect on the regulation of the formation of primary cilia and the IHH pathway.

### The Relationship Between Yes-Associated Proteins and Primary Cilia in the Chondrogenesis of Synovial Mesenchymal Stem Cell Regulated by Microtubule Stabilization

**FIGURE 5:**
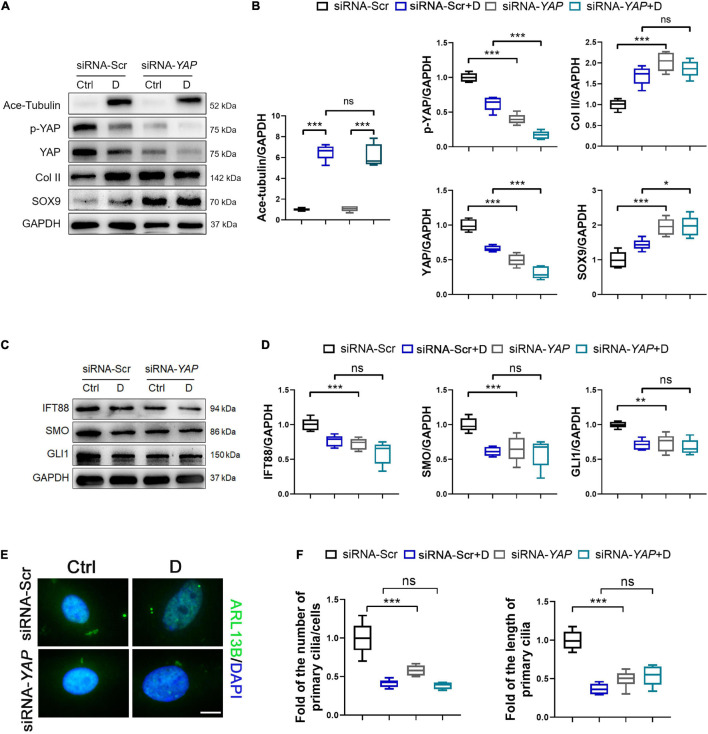
Knockdown of YAP promotes chondrogenesis and inhibits primary cilia formation in SMSCs. **(A)** Western blot analysis of Ace-Tubulin, phosphorylated Yes-associated protein (YAP), YAP, Col II, and SOX9 in synovial mesenchymal stem cells (SMSCs) transfected with siRNA-YAP and treated with docetaxel (D) for 1 week. **(B)** Quantification of the data of **(A)**. *n* = 5. **(C)** Western blot analysis of IFT88, SMO, and GLI1 in SMSCs transfected with siRNA-YAP and treated with docetaxel (D) for 1 week. **(D)** Quantification of the data of **(C)**. *n* = 5. **(E)** Immunofluorescence staining for ARL13B in SMSCs. **(F)** Quantification of **(E)** for the number and length of primary cilia in SMSCs. *n* = 5. Data are represented as the mean ± SEM. *p < 0.05, **p < 0.01, ***p < 0.001.

**FIGURE 6:**
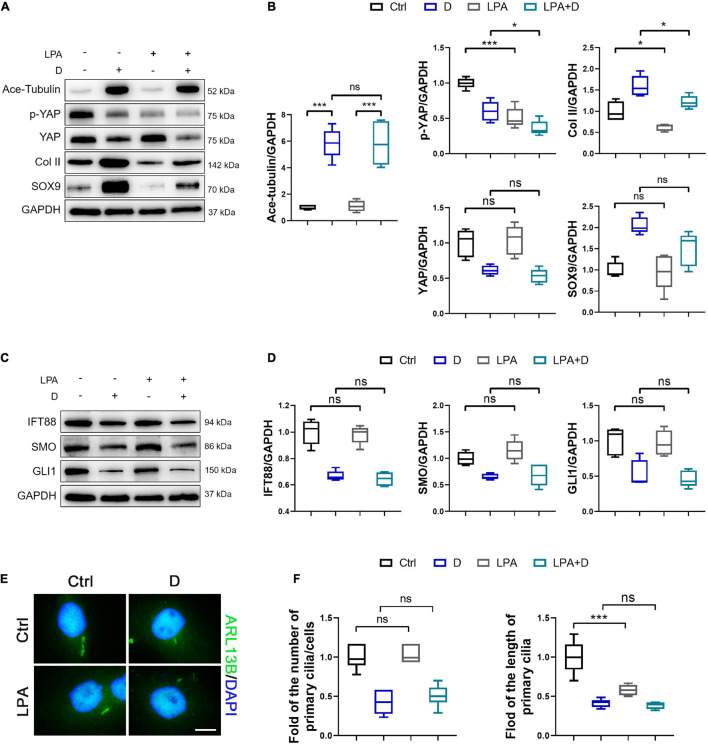
Activation of YAP diminishes chondrogenesis and cilium elongation in SMSCs. **(A)** Western blot analysis of Ace-Tubulin, phosphorylated Yes-associated protein (YAP), YAP, Col II, and SOX9 in synovial mesenchymal stem cells (SMSCs) treated with lysophosphatidic acid (LPA) and treated with docetaxel (D) for 1 week. **(B)** Quantification of the data of **(A)**. *n* = 5. **(C)** Western blot analysis of IFT88, SMO, and GLI1 in SMSCs treated with lysophosphatidic acid (LPA) and treated with docetaxel (D) for 1 week. **(D)** Quantification of the data of **(C)**. *n* = 5. **(E)** Immunofluorescence staining for ARL13B in SMSCs. **(F)** Quantification of **(E)** for the number and length of primary cilia in SMSCs. *n* = 5. Data are represented as the mean ± SEM. *p < 0.05, ***p < 0.001.

To demonstrated the role of YAP expression in chondrogenesis and primary cilia formation of SMSCs on MT stabilization, we performed the knockdown of YAP by siRNA and the promotion of nucleus re-localization of YAP by lysophosphatidic acid (LPA) through Rho GTPase activity (Yu et al., 2012). As the total and phosphorylated YAP both decreased by YAP siRNA transfection, the level of cartilage-related proteins (SOX9 and Col II) was upregulated, and the Ace-Tubulin expression was not significantly changed (Figures 5A,B). Subsequently, the expression of IFT88, SMO, and GLI1 (Figures 5C,D), and the number and the length of primary cilia (Figures 5E,F) were decreased on YAP knock-down, with or without docetaxel in SMSCs. Besides, western blot analysis showed that the decrease of the level of phosphorylated YAP by LPA treatment was detrimental for the expression of SOX9 and Col II but did not affect the Ace-Tubulin level (Figures 6A,B). However, MT stabilization by docetaxel treatment in SMSCs still had a slight effect in improving the expression of SOX9 and Col II when treated by LPA (Figures 6A,B). The LPA treatment to SMSCs did not influence the expression of IFT88 and IHH pathway, while they were inhibited in the LPA+docetaxel group due to the inhibition of MT stabilization driven by YAP expression (Figures 6C,D). The alteration in the phosphorylation of YAP via LPA treatment changed the length of primary cilia without affecting the formation of primary cilia (Figures 6E,F). Therefore, the chondrogenesis effect induced by MT stabilization was mainly through the regulation on YAP, and the expression of YAP was necessary for primary cilia formation.

## Discussion

**FIGURE 7:**
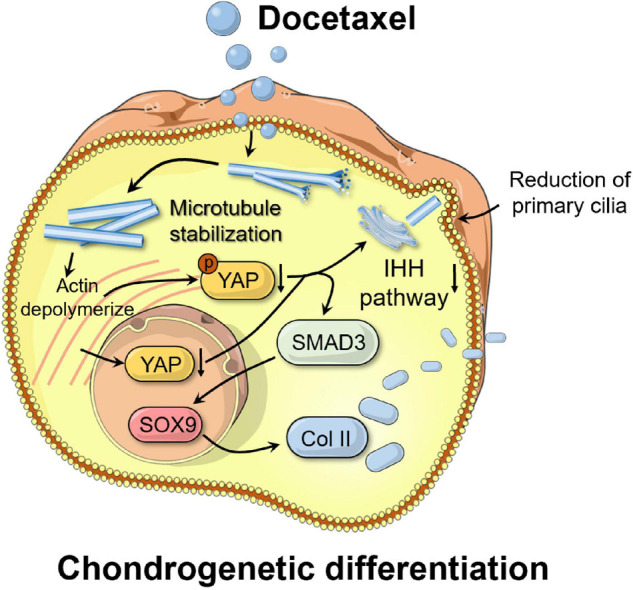
Schematic of the mechanism by which microtubule stabilization potentiates chondrogenic differentiation of synovial mesenchymal stem cells. By inhibiting Yes-associated protein (YAP) expression and activity, stabilized microtubule (MT) activated the chondrogenesis. MT stabilization repressed primary cilia formation, inhibited the Indian hedgehog (IHH) signal, and promoted chondrogenic differentiation of SMSCs.

This study demonstrated that MT stabilization had a significant effect in enhancing the chondrogenic differentiation of SMSCs (Figure 7). By inhibiting YAP expression and activity, stabilized MT activated the TGF-β/SMAD pathway and enhanced the expression of SOX9 and Col II. MT stabilization repressed primary cilia formation, inhibited the IHH signal, and promoted chondrogenic differentiation of SMSCs.

MTs play a role as “railways” for intracellular transport, and interact with several proteins to assemble to larger and intact structures (Goodson and Jonasson, 2018). In most cell types, MT displays a dynamic instability, in which the ends of individual polymers transition randomly between periods of growth and shortening (Goodson and Jonasson, 2018). The dynamic nature of the MTs allows cells to adapt to changes in cell shape and environment. However, the excessive turnover of MTs might seem wasteful in energy. Furthermore, stabilization of dynamic MTs provides a mechanism for responding to internal or external signals (Kirschner and Mitchison, 1986). Acetylation of MTs protected them from mechanical breakage and represented the statues of the stability of MT (Xu et al., 2017). Ace-Tubulin were mainly bundled and polymerized that allowed the augment of kinesin run distances and protein transport. The drugs docetaxel and nocodazole are known for their effect on MT stabilization and destabilization, respectively (Cao et al., 2018). We found that the MT stability of SMSCs with docetaxel treatment increased in correlation with the enhancement of cartilage-related proteins, SMAD3 signaling, and the promotion of chondrogenic differentiation. Interestingly, our results showed that MT effectively inhibited the fibrotic phenotype during the chondrogenic differential process of SMSCs. This finding suggested that MT stabilization would be a promising strategy for prevent the fibrocartilage formation and generated the hyaline cartilage in the cartilage regeneration process. Moreover, a satisfactory source of SMSCs will strengthen the effect of SMSCs in cartilage therapeutic process. In bioinformatic results, although the synovium in the knee joint of OA donors had higher stem cell potential, their metabolism was activated. For more effective utilization of SMSCs in the clinic, further research about the characterization of SMSCs from different sources (species, basic disease, age, position of synovium, etc.) is necessary.

YAP is an important factor in the homeostasis and maturation of chondrocytes; it promotes early chondrocyte proliferation, but inhibits chondrogenesis through the TGF-β/SMAD signaling (Ferrigno et al., 2002; Karystinou et al., 2015; Deng et al., 2016). Mechanical forces between cells or cell and ECM, and the cytoskeleton are the main factors to regulate the activity and expression of YAP. However, the role of MT stabilization in regulating YAP, especially in the differentiation of SMSCs, was still elusive before. In our results, the expression of YAP was significantly inhibited in SMSCs because of increased MT stability and the further depolymerization of actin. We investigated the two opposite upstream signaling pathways of YAP, the Hippo pathway that acts as a negative regulator and the RhoA/Rock pathway that acts as a positive regulator. The increased Hippo pathway activity and inhibition of the RhoA/Rock pathway mediated by MT stabilization *in vivo* supported the increased ratio of phosphorylated YAP in total observed in SMSCs. Moreover, the chondrogenic effect induced by MT stabilization in SMSCs was through the repression of YAP, which was identified by the YAP knockdown by siRNA and treatment with the YAP agonist, LPA. A recent study revealed that YAP has a reciprocal antagonistic effect on NF-κB signaling that results in reduced matrix-degrading enzyme expression and cartilage degradation during OA (Deng et al., 2018). In addition, connective tissue growth factor (CTGF) was identified as a direct YAP target (Zhao et al., 2008), which was responsible for promoting Col I synthesis and fibrous tissue formation (Lee et al., 2014). Therefore, in cartilage injury repair, the YAP activity in the early stage could ensure proliferation of chondrocytes and their resistance to inflammatory factors. However, its continued existence would contribute to formation of fibrocartilage rather than hyaline cartilage due to the promotion of CTGF activity and inhibitory effect of TGF-β on YAP. Collectively, these findings suggest that MT stabilization inhibits YAP activity to drive a cartilaginous phenotype and chondrogenic differentiation of SMSCs.

In this study, stabilized MT in SMSCs mainly displayed as a form of primary cilia, which was responsible for biomechanical signal sensing and substance transportation. Moreover, the main complex and components of IHH signaling pathway are located at the primary cilia, which is involved in the regulation of chondrocyte hypertrophy and osteogenic differentiation through RUNX2 and Wnt/β-catenin signaling pathway (Katoh, 2007; Bangs and Anderson, 2017). In SMSCs, MT stabilization reduced primary cilia formation and inhibited the IHH pathway with further osteogenic differentiation. Knock-down of YAP decreased the formation of primary cilia. However, LPA treatment enhanced the re-localization of YAP to the nucleus and led to the shortening of primary cilia but did not change the activity of the IHH pathway. We observed that the IHH pathway was attenuated in SMSCs by MT stabilization, which suggested that the level of IHH pathway activity was mainly affected by the quantity of primary cilia rather than their length. It was reported that cytoplasmic retention of YAP correlated with active ciliogenesis in human retinal pigmented epithelial cells (Kim et al., 2015). However, in pronephros development in zebrafish, YAP was necessary for ciliogenesis and morphogenesis (He et al., 2015). We speculated that YAP is required for formation of primary cilium even though increased YAP activity inhibits its elongation. Moreover, although primary cilia might be not beneficial during the chondrogenic differentiation of MSCs, it was an important structure for the function of mechanical transduction in chondrocytes. Herein, more specific research about the role of MT stabilization in the regulation primary cilia and YAP during the transformation from MSC to chondrocyte remain to be elucidated.

Taken together, our study highlights a novel mechanism for chondrogenesis of SMSCs and presents a promising therapeutic target for articular cartilage regeneration. First, drugs that affect the stability and polymerization of MTs have been extensively studied, and some have been commercialized (Cao et al., 2018) and can be directly used as candidates for treating cartilage injury or exploring its underlying mechanism. Then, there are many signaling pathways, including PI3K-Akt, ERK1/2, RhoA, CDC42, Rock, and RTKs (Roostalu and Surrey, 2017; Stanganello et al., 2019; Moujaber and Stochaj, 2020), as well as MT stabilization associated proteins, including Tau, MAPs, and fibroblast growth factor 13 (FGF13) (Wu et al., 2012; Prezel et al., 2018; Melkova et al., 2019), that can directly or indirectly affect MT stability, making this class of molecules potential therapeutic targets. We believe that MT stabilization is a profound mechanism and target in the therapy of cartilage disease.

## Data Availability Statement

The original contributions presented in the study are included in the article/Supplementary Material, further inquiries can be directed to the corresponding author/s.

## Ethics Statement

The studies involving human participants were reviewed and approved by the Ethical Committee of the Nanjing Drum Tower Hospital, the Affiliated Hospital of Nanjing University Medical School. The patients/participants provided their written informed consent to participate in this study.

## Author Contributions

DS and JL: conceptualization. JL, ZL, ZS, MW, HJ, GT, AL, HS, XX, and WY: methodology. JL, DS, SI, ZL, and ZS: visualization. DS and QJ: funding acquisition. DS, RW, and QJ: project administration. DS, QJ, and SI: supervision. JL: writing—original draft. DS, SI, and JL: writing—review and editing. All authors contributed to the article and approved the submitted version.

## Conflict of Interest

The authors declare that the research was conducted in the absence of any commercial or financial relationships that could be construed as a potential conflict of interest.

## Publisher’s Note

All claims expressed in this article are solely those of the authors and do not necessarily represent those of their affiliated organizations, or those of the publisher, the editors and the reviewers. Any product that may be evaluated in this article, or claim that may be made by its manufacturer, is not guaranteed or endorsed by the publisher.

## Footnotes

1
http://www.ncbi.nlm.nih.gov/geo/
