# Supplementary material for: Microtubule Stabilization Enhances the Chondrogenesis of Synovial Mesenchymal Stem Cells
Source: Front Cell Dev Biol. 2021 Oct 20;9:748804. doi: 10.3389/fcell.2021.748804 (PMC8564364; doi:10.3389/fcell.2021.748804)
Supplement: Supplementary file 1 [file Data_Sheet_1.PDF]

Supplementary information

Microtubule stabilization enhances the chondrogenesis of synovial mesenchymal stem cells

Jiawei Li[1], Ziying Sun[1], Zhongyang Lv[1], Huiming Jiang[3], Anlong Liu[1], Maochun Wang[1], Guihua Tan[1], Hu Guo[1], Heng Sun[1], Rui Wu[1], Xingquan Xu[1], Wenjin Yan[1], Qing Jiang[1], Shiro Ikegawa[1,4], Dongquan Shi[1]*

SOX9	Forward primer	5’-AGCGAACGCACATCAAGAC-3’
	Reverse primer	5’-CTGTAGGCGATCTGTTGGGG-3’
COL2A1	Forward primer	5’-CCAGATGACCTTCCTACGCC-3’
	Reverse primer	5’- TTCAGGGCAGTGTACGTGAAC-3’
RUNX2	Forward primer	5’- TGGTTACTGTCATGGCGGGTA-3’
	Reverse primer	5’- TCTCAGATCGTTGAACCTTGCTA-3’
COL1A1	Forward primer	5’- GAGGGCCAAGACGAAGACATC-3’
	Reverse primer	5’- CAGATCACGTCATCGCACAAC-3’
COL10A1	Forward primer	5’- ATGCTGCCACAAATACCCTTT-3’
	Reverse primer	5’- GGTAGTGGGCCTTTTATGCCT-3’
MST1	Forward primer	5’- CAGAGCTGCGGCATCAAATC-3’
	Reverse primer	5’- ACCTTGGTCGAGGAACTTGC-3’
MST2	Forward primer	5’- TCTGAGATTGTGGAGGCCATTC-3’
	Reverse primer	5’- GCTCCGTTCCTAAGGCAGAT-3’
LATS1	Forward primer	5’- CTGCTCTCCCCTCCAGAGTTA-3’
	Reverse primer	5’- TGGCAGGAAAGGTCTTAGGC-3’
LATS2	Forward primer	5’- TGCCAACAATGTAGCGAATGT-3’
	Reverse primer	5’- TTGAAGATTATCACTCTCTCCAGG-3’
RHOA	Forward primer	5’- GATTGGCGCTTTTGGGTACAT-3’
	Reverse primer	5’- AGCAGCTCTCGTAGCCATTTC-3’
ROCK1	Forward primer	5’- AAGAGGGCATTGTCACAGCA-3’
	Reverse primer	5’- AGCATCCAATCCATCCAGCA-3’
ROCK2	Forward primer	5’- CCCGATAACCACCCCTCTTC-3’
	Reverse primer	5’- TGCCTTGTGACGAACCAACTG-3’
GAPDH	Forward primer	5’-ACAACTTTGGTATCGTGGAAGG-3’
	Reverse primer	5’- GCCATCACGCCACAGTTTC-3’

Table.S1. The primer sequences used in RT-PCR analysis

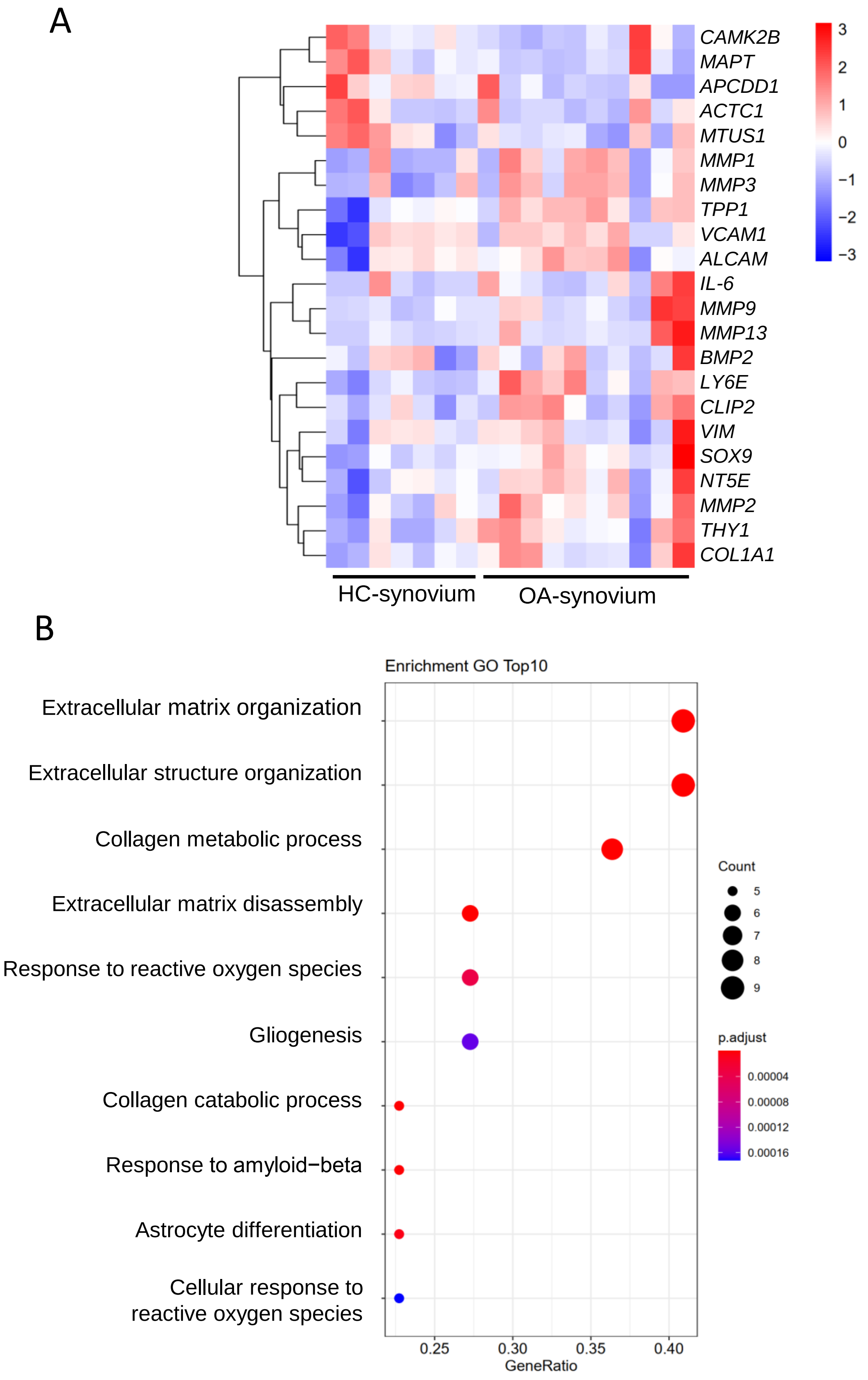

Supplementary Figure1.

(A) Heatmap and (B) gene ontogeny analyses for inflammatory genes and stem cell related marker genes in knee joint synovium from healthy (Control) and osteoarthritis (OA) donors.
